# Emergence of mcr-8.2-harboring hypervirulent ST412 Klebsiella pneumoniae strain from pediatric sepsis: A comparative genomic survey

**DOI:** 10.1080/21505594.2022.2158980

**Published:** 2022-12-25

**Authors:** Ruishan Liu, Hao Xu, Junhui Zhao, Xinjun Hu, Lingjiao Wu, Jie Qiao, Haoyu Ge, Xiaobing Guo, Jianjun Gou, Beiwen Zheng

**Affiliations:** a Collaborative Innovation Center for Diagnosis and Treatment of Infectious Diseases, State Key Laboratory for Diagnosis and Treatment of Infectious Diseases, the First Affiliated Hospital, College of Medicine, Zhejiang University, Hangzhou, China; b Department of Laboratory Medicine, The First Affiliated Hospital of Zhengzhou University, Zhengzhou, China; c Jinan Microecological Biomedicine, Shandong Laboratory, Jinan, China; d School of Basic Medical Sciences, Zhejiang Chinese Medical University, Hangzhou, China; e Department of Infectious Diseases, The First Affiliated Hospital, College of Clinical Medicine, Henan University of Science and Technology, Luoyang, China; f Research Units of Infectious Diseases and Microecology, Chinese Academy of Medical Sciences, Hangzhou, China

**Keywords:** MCR-8, colistin resistance, *Klebsiella pneumoniae*, paediatric sepsis, hypervirulent

## Abstract

Emerging mobile colistin resistance (*mcr*) genes pose a significant threat to public health for colistin was used as the last resort to treat multidrug-resistant (MDR) pathogenic bacterial infections. Hypervirulent *Klebsiella pneumoniae* (hvKP) is a clinically significant pathogen resulting in highly invasive infections, often complicated by devastating dissemination. Worryingly, the untreatable and severe infections caused by *mcr*-harbouring hvKP leave the selection of antibiotics for clinical anti-infective treatment in a dilemma. Herein, we screened 3,461 isolates from a tertiary teaching hospital from November 2018 to March 2021, and an *mcr-8.2*-harbouring hvKP FAHZZU2591 with a conjugative plasmid was identified from paediatric sepsis. This is the first report of MCR-8-producing hvKP from paediatric sepsis to our best knowledge. The susceptibility, genetic features, and plasmid profiles of the isolate were investigated. Further, we assessed the virulence potential of FAHZZU2591 and verified its pathogenicity and invasive capacity using a mouse model. The phylogenetic analysis of *mcr-8*-bearing *K. pneumoniae* revealed that China is the predominant reservoir of the *mcr-8* gene, and the clinic is the primary source. Our work highlights the risk for the spread of *mcr*-positive hvKP in clinical, especially in paediatric sepsis, and the persistent surveillance of colistin-resistance hvKP is urgent.

## Introduction

Increasing reports of colistin resistance led to global attention, for colistin was used as the last resort to treat multidrug-resistant (MDR) pathogenic bacterial infections. However, the emergence of colistin-resistant *Klebsiella pneumoniae* brings great difficulty to clinical treatment. The resistance mechanism to colistin is mainly due to the modification of lipid A, the anchor of the lipopolysaccharide (LPS) [1]. Both chromosomes and plasmids encoded the modifications. Chromosome-encoded resistance is most commonly regulated by mutations in two-component systems such as PmrA/PmrB or PhoP/PhoQ, or alterations to the regulator MgrB [2]. In contrast, plasmid-encoded resistance is realized by the expression of MCR-type phosphoethanolamine transferase [3].

The mobilized colistin resistance determinants (*mcr*) indicate that colistin resistance is no longer limited to vertical transmission but can spread horizontally among different species. To date, ten *mcr* genes and some variants have been identified across the globe [4]. Among these, *mcr-1* is the most widely disseminated. At the same time, the *mcr-8* gene has rarely been reported and is mainly found in *K. pneumoniae* and closely related species (*Klebsiella quasipneumoniae*, *Raoultella ornithinolytica*) [3,5]. So far, five variants of the *mcr-8* gene have been identified (*mcr-8.1* to *mcr-8.5*), and *mcr-8.2* was primarily present in animals and environment-related isolates [5–7]. Worryingly, in China, *mcr-8.2* has been detected in various types of clinical specimens across different cities [8–10]. Of note, only two reports described the *mcr-8.2*-bearing strains with conjugative plasmids worldwide [9,10].

Hypervirulent *K. pneumoniae* (hvKP) was first described in 1986 in Taiwan, which caused a clinical syndrome of community-acquired *K. pneumoniae* infections [11,12]. hvKP could infect healthy individuals of any age. The infections usually involve multiple sites and clonal spread [13]. Though hvKP rarely exhibits a resistant phenotype, the antibiotic-resistant hvKP is increasing with the widespread of antibiotic-resistance genes. Notably, the emergence and transmission of *mcr*-positive hvKP would become a serious public problem. Bacteraemia is the leading cause of infectious disease morbidity and mortality worldwide in children [14,15]. Compared with the mortality of pneumonia, the mortality caused by *K. pneumoniae* bloodstream infection (Kp-BSI) in young children is even higher [16]. However, studies related to *mcr*-harbouring hvKP involving paediatric sepsis are still lacking.

In this work, we did a retrospective study to screen *mcr*-harbouring isolates from 3,461 strains in a tertiary teaching hospital in China from 2018 to 2021. And a hvKP isolate FAHZZU2591 with a conjugative *mcr-8.2*-carrying plasmid was isolated from a paediatric sepsis case. The complete sequence of conjugative *mcr-8.2*-carrying plasmid pFAHZZU2591 has been determined. In addition, we investigated the virulence features of the isolate and performed the phylogenetic analyses of *K. pneumoniae* isolates harbouring-*mcr-8.2*.

## Materials and methods

### Isolates, species identification, and antimicrobial susceptibility testing

From November 2018 to March 2021, we screened *mcr*-harbouring isolates from 3,461 strains. Strains were recovered from patients submitting specimens to the First Affiliated Hospital of Zhengzhou University. Species identification was conducted using matrix-assisted laser desorption/ionization time-of-flight mass spectrometry (MALDI-TOF/MS) (Bruker Daltonik GmbH, Bremen, Germany). The *mcr* genes were identified under PCR and DNA sequencing. Specific primer sequence information and experimental conditions were showing in Table S1. Clinical data were obtained from medical record systems. Ethical approval was granted by the Ethics Committee of the First Affiliated Hospital of Zhejiang University.

The Minimum inhibitory concentration (MIC) values of strain FAHZZU2591 and transconjugant FAHZZU2591-*E. coli* 600 were determined using the agar dilution method and broth microdilution method. The results were interpreted according to the guidelines of CLSI 2020 (https://clsi.org) except for tigecycline and colistin, which were interpreted following EUCAST clinical breakpoints (https://www.eucast.org/). *Escherichia coli* ATCC 25,922 was used as quality control.

### Location of mcr-8.2 gene and transferability of the plasmid carrying mcr-8.2

S1-PFGE was conducted to determine the size and number of plasmids of *K. pneumoniae* FAHZZU2591. And the location of *mcr-8.2*, *rmpA*, and *rmpA2* were identified by Southern blotting and hybridization with digoxigenin-labelled specific probes (Table S2). Briefly, DNA plugs with whole-cell genomic DNA of culture-lysed cells (FAHZZU2591, *E. coli* 600, and transconjugant FAHZZU2591-*E. coli* 600) were digested using the restriction enzyme S1 (Takara Bio Inc., Japan). *Salmonella enterica* serotype Braenderup H9812 was used as a size marker and digested using the restriction enzyme XbaI. S1-PFGE was undertaken on a CHEF-DR III system (Bio-Rad, Hercules, CA, USA) using the following parameters: running time 18 h, temperature 14℃, field strength 6 V/cm^2^, initial pulse time 2.2 s, final pulse time 63.8 s. The upward capillary transfer was used to transfer DNA from agarose gel to a positively charged nylon membrane. Southern hybridization was conducted with the DIGHigh Prime DNA Labelling and Detection Starter Kit II (Roche Diagnostics). The digoxigenin-labelled probes (*mcr-8.2*, *rmpA*, and *rmpA2*) are used to hybridize with membranes, and the plasmids or chromosomes carrying the target genes are mapped after colour development.

Conjugation experiments were carried out with rifampicin-resistant *E. coli* 600 as a recipient strain. Then the transconjugants were selected on a Mueller-Hinton agar (OXOID, Hampshire, United Kingdom) medium containing 200 mg/L rifampicin and one mg/L colistin. Finally, a combination of MALDI-TOF/MS identification and *mcr-8.2* gene detection was performed to confirm the plasmid was successfully transferred to the recipient.

### Whole-genome sequencing (WGS) and in silico analyses

All of the MCR-positive isolates were analysed by WGS. DNA was extracted using a Bacterial DNA Kit (QIAGEN, Hilden, Germany). Then the library construction was performed using a 350 bp small fragments genomic DNA library. The whole genome of *K. pneumoniae* FAHZZU2591 was subsequently sequenced using the Nanopore PromethION platform (Oxford Nanopore Technologies, Oxford, United Kingdom) and Illumina NovaSeq 6000 (Illumina, San Diego, CA, United States). After sequencing, the short and long reads were hybrids assembled with Unicycler v0.4.7 to get the complete genome sequence [17]. Additionally, the genomic sequence was annotated using Prokka, while the IS elements and transposon were identified by ISfinder (http://www-is.biotoul.fr/). The sequence was deposited in the database of Institute Pasteur (http://bigsdb.web.pasteur.fr/klebsiella/klebsiella.html) to confirm the capsular serotype and multilocus sequence type. And the replicon type of plasmid and acquired antimicrobial resistance genes were determined using online tools (http://www.genomicepidemiology.org/). Finally, the comparison of genetic environments surrounding *mcr-8.2* genes on various plasmids was performed using Easyfig 2.2.3 [18]. The circular map of multiple plasmid comparisons was generated with the BLAST Ring Image Generator (BRIG) [19].

### Identification of hvKP

According to a recent study, the use of five genotypic markers, including the plasmid-borne *rmpA* gene (_p_rmpA, _p_rmpA2), salmochelin siderophore biosynthesis (*iroB*), aerobactin siderophore biosynthesis (*iucA*) and putative transporter (*peg-344*) was shown to differentiate hvKP from cKp strains, all of which achieved a diagnostic accuracy ≥0.95 [20]. Thus, we use Institute Pasteur (http://bigsdb.pasteur.fr/klebsiella/klebsiella.html) to identify the virulence genes of isolate in this study to confirm virulence-associated features and defined hvKP.

### Virulence assessment with serum killing and Galleria mellonella infection assays

The serum-killing assay was used to evaluate the survivability of the isolates exposed to healthy human serum. Blood was drawn from healthy humans and left at 37 ℃ for one hour, then centrifuged at a speed of 4000 rpm/min for eight minutes to collect the upper serum. The serum was divided into two parts, and one part was placed at 56℃ for 30 min to inactive complement. Overnight bacterial culture was diluted to a concentration of 1 × 10^6^ CFU/ml, and 20 µl of bacterial suspension plus 180 µl inactivated serum or normal serum at 37 ℃ for one hour. The incubated samples were placed on ice to terminate the reaction, then were coated on Mueller – Hinton agar plates and counted colonies after overnight culture. The bacterial survival rate was calculated using the following formula: Bacterial survival rate = (number of colonies with normal serum/number of colonies with inactivated serum) × 100%. Results were analysed with the unpaired *t* test. The experiment was conducted in triplicate, and *K. pneumoniae* ATCC 700,603 was used as the negative control [21].

Overnight cultures of isolates were washed with PBS and diluted to 1 × 10^6^ CFU/ml. *Galleria mellonella* larvae were randomly divided into groups of ten, and each larva was injected with 20 µl diluted bacteria. *K. pneumoniae* ATCC 700,603 and PBS were used as low virulence control and negative control group, respectively. And the transconjugant FAHZZU2591-*E. coli* 600 and recipient strain *E. coli* 600 were used to prove the contribution of plasmid pFAHZZU259 on virulence. The number of surviving larvae was first recorded at the eighth after injection and then recorded every twelve hours. The experiment was performed in triplicate for each isolate. The survival rate was calculated as the mean ratio of surviving larvae [21]. The Kaplan-Meier estimator method was used to plot a survival curve for *G. mellonella*. And Survival curves were compared with the log-rank (Mantel – Cox) test.

### Mouse infection model

Animal assays were carried out under the guidelines for the Care and Use of Laboratory Animals of the Chinese Association for Laboratory Animal Sciences. The mouse infection model was established using 5-week-old female BALB/C mice (Shanghai Model Organisms Center, Inc., Shanghai, China) and housed for a week before infection (each group injected four mice respectively). Each mouse in the experimental group was injected with 100 µl 1 × 10^8^ bacterial cells suspended (FAHZZU2591). The low virulence control and negative control were conducted using *K. pneumoniae* ATCC 700,603 and normal saline (100 μl). Monitored and recorded the physical condition of each mouse. All surviving mice were euthanized after seven days of injection. The ileum and lung tissues were dissected, fixed in 10% formalin, and embedded in paraffin. Sections were cut and stained with haematoxylin and eosin. Faeces of the mice were collected on the first, third, fifth, and seventh days after injection before the mice were euthanized. Take equal amounts of collected faecal samples and homogenized them in 1 ml of normal saline. Then the homogenized tissue was diluted 100 times and plated on blood agar. Strains isolated from the faeces of mice were under PCR screening (*mcr-8*, *rmpA*, and *rmpA2*) and species identification. *K. pneumoniae* strains with positive results were selected for homology verification with FAHZZU2591 by PFGE.

### Phylogenetic analysis

To investigate the phylogenetic relationships among *K. pneumoniae* strains carrying *mcr-8*, we downloaded 16,472 publicly available *K. pneumoniae* genomes from the NCBI database. Then, 74 genomes were identified carrying the *mcr-8* gene by resfinder. These genomes, including the FAHZZU2591 genomic sequence, were then performed phylogenetic analyses using Roary [22]. Subsequently, a maximum likelihood phylogenetic tree based on core genes identified by Roary was generated using MEGA X. The modification and visualization were performed by iTOL (https://itol.embl.de/).

### Data availability

The complete genome sequence of *K. pneumoniae* FAHZZU2591 was deposited to GenBank with the accession numbers CP083751-CP083753.

## Results

### Isolation and identification of mcr-8.2-producing K. pneumoniae FAHZZU2591 strain

**Figure 1.**
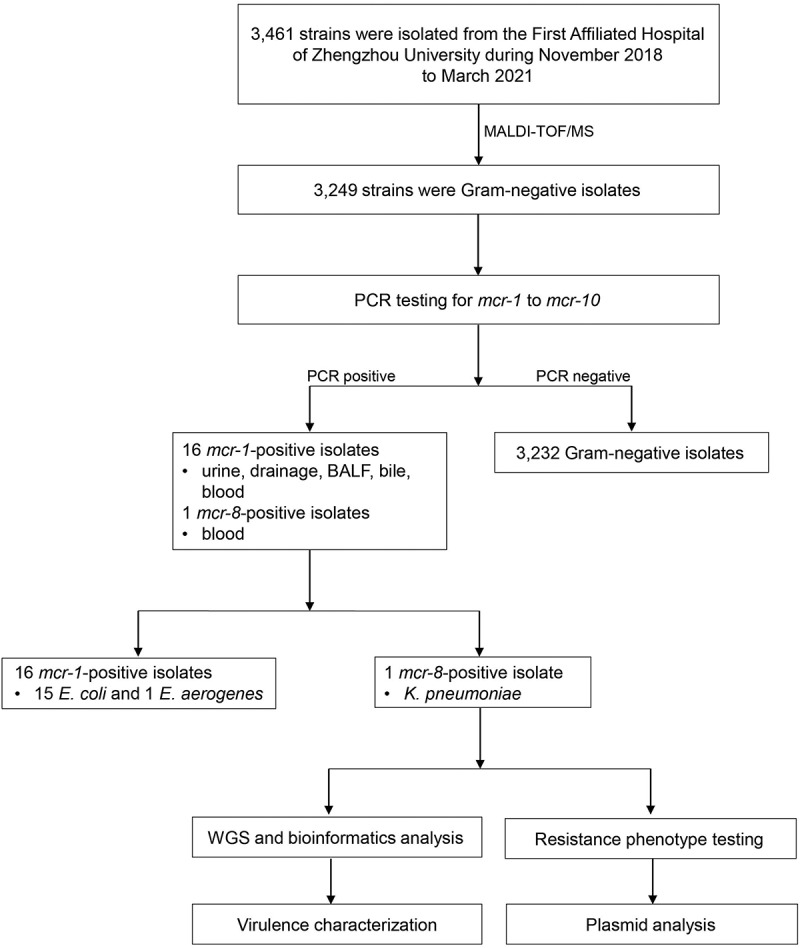
Schematic procedure of the study. MALDI-TOF/MS, matrix-assisted laser desorption/ionization time-of-flight mass spectrometry; BALF, Bronchoalveolar Lavage Fluid; hvKP, hypervirulent *Klebsiella pneumoniae*; *E. coli*, *Escherichia coli*; *E. aerogenes*, *Enterobacter aerogenes*; WGS, whole-genome sequencing.

We collected 3,461 isolates from 62 departments of the First Affiliated Hospital of Zhengzhou University from 2018 to 2021 (Figure 1). Among them, 3,249 strains were Gram-negative and underwent PCR testing of *mcr* genes. The results showed that sixteen isolates (fifteen *E. coli* and one *Enterobacter aerogenes*) were *mcr-1*-positive while only one isolate (designated as FAHZZU2591) was *mcr-8*-harbouring. No other *mcr* genes were detected. FAHZZU2591 was isolated from a 1-year-old female outpatient of the Department of Hepatobiliary Extrapancreatic and Liver Transplantation in September 2020. The patient underwent a routine examination and subsequently was diagnosed as sepsis. *K. pneumoniae* FAHZZU2591 was recovered from the blood culture. Subsequently, the isolate was identified as *K. pneumoniae* using MALDI-TOF/MS and 16S rRNA sequencing and was found to carry *mcr-8.2* gene after PCR and sequencing.

### Antimicrobial susceptibility profiles

As shown in Table 1, the results of antibiotic susceptibility testing indicated *K. pneumoniae* FAHZZU2591 was resistant to colistin with a MIC value of four mg/L. In addition, the strain also exhibited resistance to ceftriaxone, cefepime, and cefotaxime, and was intermediate to amoxicillin/clavulanate, ceftazidime, and aztreonam. It was shown to be susceptible to piperacillin/tazobactam, ciprofloxacin, levofloxacin, imipenem, meropenem, trimethoprim/sulfamethoxazole, amikacin, gentamicin, chloramphenicol, and tigecycline. Moreover, the transconjugant FAHZZU2591-*E. coli* 600 exhibited a similar resistance profile to FAHZZU2591. FAHZZU2591-*E. coli* 600 also showed resistance to colistin, and the MIC value was four mg/L.

**Table 1.** MIC values of antimicrobials for *K. pneumoniae* FAHZZU2591, transconjugant FAHZZU2591-*E. coli 600* and recipient strain *E. coli* C600.

	MIC values (mg/L)		
Antimicrobials	*K. pneumoniae* FAHZZU2591	FAHZZU2591-*E. coli* 600	*E. coli* 600
Amoxicillin/clavulanate	16/8	16/8	8/4
Piperacillin/tazobactam^a^	2/4	2/4	4/4
Ceftazidime	8	8	0.5
Ceftriaxone	64	64	≤0.03
Cefepime	16	16	≤0.008
Cefotaxime	64	64	0.03
Ciprofloxacin	0.25	0.25	0.25
Levofloxacin	0.5	0.5	0.25
Imipenem	0.125	0.5	0.5
Meropenem	0.03	0.03	0.03
Trimethoprim/ sulfamethoxazole	0.125/2.375	0.125/2.375	0.125/2.375
Amikacin	2	2	2
Gentamicin	1	0.5	0.5
Aztreonam	8	8	0.125
Chloramphenicol	4	4	4
Colistin	4	4	1
Tigecycline	0.25	0.06	≤0.03

^a^Tazobactam at a fixed concentration of 4 mg/L.

### Molecular characteristics of *mcr-8*-harbouring hvKP FAHZZU2591

The genomic sequence of strain *K. pneumoniae* FAHZZU2591 indicates it belongs to ST412 and capsule type *wzi*206/K57. The complete genome of FAHZZU2591 consists of a ~ 5.2Mb circular chromosome, a ~ 202kb pLVPK-like virulent plasmid (designated as pFAHZZU2591), and a ~ 110kb *mcr-8.2*-harboured plasmid (designated as pFAHZZU2591mcr-8). The average G + C content of chromosomes and plasmids was 57.6%, 49.9%, and 52.0%, respectively. Further, the chromosome contained 4,787 protein-coding genes, 85 tRNAs, and 25 rRNAs (Table S3). Based on the results of Resfinder, a total of eight antimicrobial resistance genes were identified, including beta-lactamase genes (*bla*_SHV-1_, *bla*_TEM-1B_, and *bla*_CTX-M-3_), quinolone resistance gene (*qnrS1*), fosfomycin resistance gene (*fosA*), efflux pump-associated genes (*oqxB* and *oqxA*) and mobile colistin resistance gene (*mcr-8.2*), of which four were encoded on plasmid pFAHZZU2591mcr-8 (Table 2).

**Table 2.** Antibiotic resistance genes of *K. pneumoniae* FAHZZU2591.

Genome	Drug class	Coding genes	Position
Chromosome	Efflux pump-associated gene	*oqxB*	1,163,662–1,166,814
	Efflux pump-associated gene	*oqxA*	1,166,838–1,168,013
	Beta-lactam	*bla*_SHV-1_	2,680,596–2,681,456
	Fosfomycin	*fosA*	4,553,820–4,554,162
pFAHZZU2591mcr-8	Colistin	*mcr-8.2*	12,162–13,859
	Beta-lactam	*bla*_TEM-1B_	29,931–30,791
	Beta-lactam	*bla*_CTX-M-3_	31,573–32,448
	Quinolone	*qnrS1*	37,500–38,156

### Plasmid profiles and virulence-associated features

Strain *K. pneumoniae* FAHZZU2591 contains three virulence-gene clusters, one of which is sited on the chromosome (type 3 fimbriae gene cluster *mrkABCDFHIJ*). At the same time, the other two are located on the pLVPK-like virulent plasmid pFAHZZU2591 (Table 3). Significantly, the two virulence gene clusters encoding siderophores (including aerobactin-encoded gene *iutAiucABCD* and salmochelin-encoded gene *iroBCDN*) and polysaccharide virulence genes (*rmpA* and *rmpA2*) lying in the pFAHZZU2591 frequently confers hypervirulent phenotype in *K. pneumoniae* [23].

**Table 3.** The detection of virulence genes in *K. pneumoniae* FAHZZU2591 based on whole-genome sequence.

Locus	Length	Site	Start position	End position
*iroB*	1116	pFAHZZU2591	22728	23843
*iroC*	3726	pFAHZZU2591	18941	22666
*iroD*	1230	pFAHZZU2591	17607	18836
*iroN*	2175	pFAHZZU2591	14973	17147
*iucA*	1791	pFAHZZU2591	120529	122319
*iucB*	948	pFAHZZU2591	122320	123267
*iucC*	1734	pFAHZZU2591	123267	125000
*iucD*	1278	pFAHZZU2591	125004	126281
*iutA*	2202	pFAHZZU2591	126363	128564
*mrkA*	609	Chromosome	830542	831150
*mrkB*	702	Chromosome	831246	831947
*mrkC*	2487	Chromosome	831959	834445
*mrkD*	996	Chromosome	834436	835431
*mrkF*	636	Chromosome	835445	836080
*mrkH*	705	Chromosome	837565	838269
*mrkI*	585	Chromosome	836975	837559
*mrkJ*	717	Chromosome	836115	836831
*rmpA*	633	pFAHZZU2591	12008	12640
*rmpA2*	636	pFAHZZU2591	133452	134087

**Figure 2.**
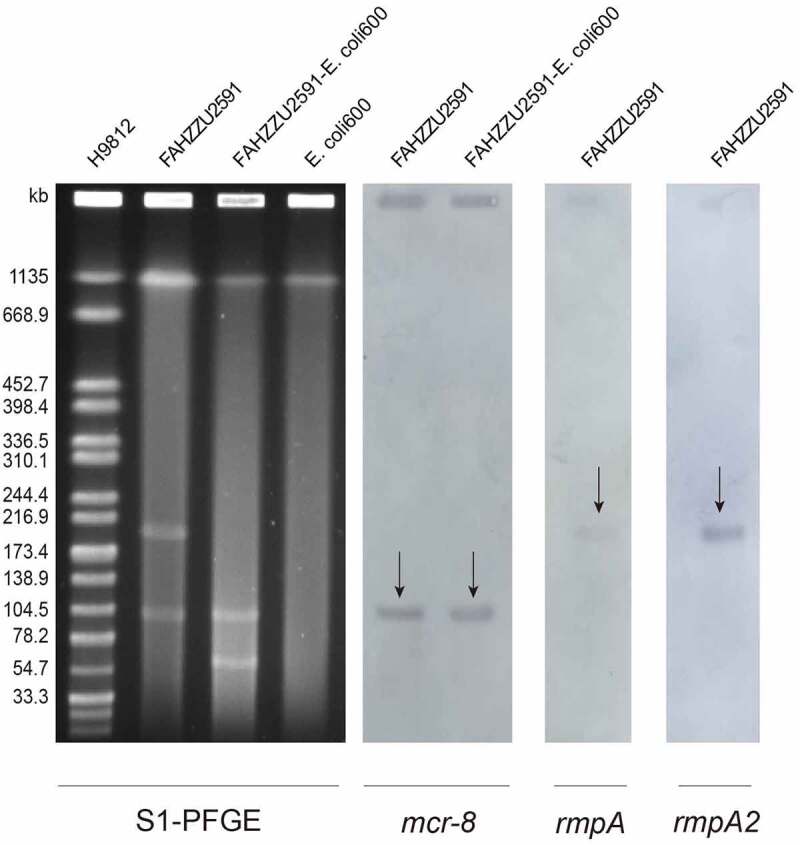
Plasmid profiles of *K. pneumoniae* FAHZZU2591, transconjugant FAHZZU2591-*E. coli* 600 and recipient strain *E. coli* 600. Plasmid size determination by S1-PFGE, with *Salmonella enterica* serotype Braenderup H9812 as the size marker. The names of the isolates are shown in the first line. The arrows in indicated the locations of *mcr-8*, *rmpA* or *rmpA2* harbouring plasmids according to the Southern blotting experiment. .

**Figure 3.**
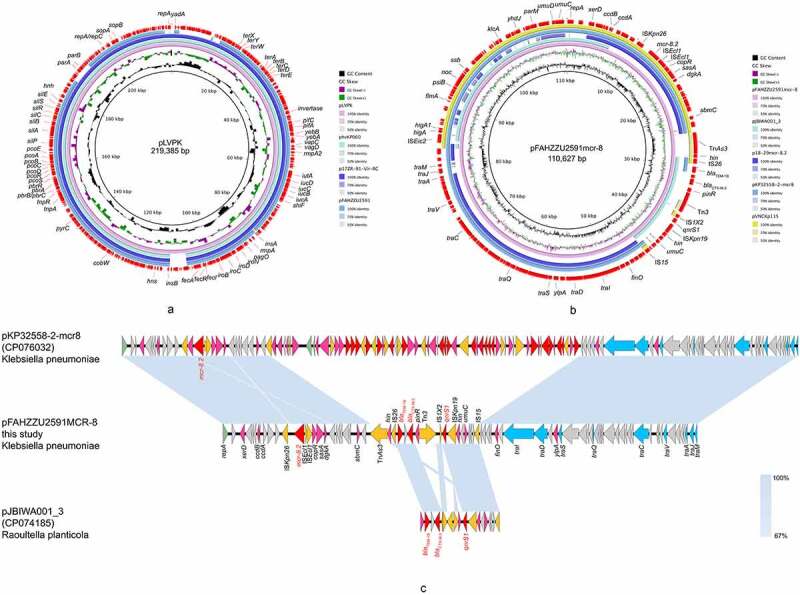
The genomic analysis of *K. pneumoniae* FAHZZU2591. (**a**) Comparison between plasmid pFAHZZU2591 and other pLVPK-like virulence plasmids based on BLASTn analyses (GenBank accession numbers from inside to outside are AY378100, CP034776, MN200128, and CP083752). Plasmid pLVPK (the outer circle) was used as the reference. (**b**) Comparison between plasmid pFahzzu2591mcr-8 and four similar plasmids in NCBI nr/nt database (accession numbers: CP074185.1, MK262711.1, CP076032.1 and LC549807.1). pFahzzu2591mcr-8 (the outer circle) was used as the reference. The different colours indicate different plasmids and are listed in the colour key. (**c**) a linear demonstration of main structural features of plasmid pFahzzu2591mcr-8 compared with plasmids pKP32558-2-mcr8 (CP076032) and pJBIWA001_3 (CP074185). Open reading frames (ORFs) are indicated as arrows that show the orientation of coding sequence with the gene name. Yellow indicates genes related to mobile elements, red indicates genes related to drug resistance, blue indicates genes involved in conjugation and pink represents other functional genes. Hypothetical protein encoded genes are coloured by grey. Regions with a high degree of homology are indicated by pale blue shading.

Consistent with genomic features, S1-PFGE and Southern-blot confirmed the size of two plasmids and the location of *mcr-8.2*-bearing plasmid pFAHZZU2591 (Figure 2). The plasmid pFAHZZU2591 belonged to the replicon type of *repB* and comprised 199 predicted coding sequences (Table S3). A BLASTN search in the NCBI nucleotide database revealed that pFAHZZU2591 showed high homology to the ~219 kb classical virulence plasmid pLVPK (accession number: AY378100) with 99.95% identity and 94% coverage. Thus, pFAHZZU2591 was considered a pLVPK-like plasmid. Compared pFAHZZU2591 to plasmid pLVPK (accession number: AY378100) and other two pLVPK-like virulence plasmids phvKP060 (accession number: CP034776) and p17ZR-91-Vir-RC (accession number: MN200128) recovered from the clinic, these four plasmids shared a similar backbone (Figure 3a) with the identity of 99.95%, 99.99%, and 99.99%, respectively.

According to the result of Plasmidfinder analysis, plasmid pFAHZZU2591mcr-8 was untypeable, for no hit was found in the database. Further analysis indicates that pFAHZZU2591mcr-8 contains two main regions: a ~ 94kb backbone region that exhibited 99% nucleotide identity to pKP32558-2-mcr8 (accession number: CP076032.1) from *K. pneumoniae* and a ~ 16kb drug resistance region which including antibiotic resistance genes, insertion sequences (ISs) and transposons was highly homologous to pJBIWA001_3 (accession number: CP074185.1) from *Raoultella planticola* (Figure 3b &amp ; 3C). The drug resistance region had two resistance units (Tn*As3*-IS*26-bla*_TEM-1B_-*bla*_CTX-M-3_-Tn*3* and IS*1×2-qnrS1*-IS*Kpn19*-IS*15*) encode the resistance to beta-lactam and fluoroquinolone antibiotics. In addition, the backbone of pFAHZZU2591mcr-8 carried a conjugal transfer region (*traACDIJMQSV*) which could promote the horizontal transfer of the plasmid among bacteria. Thus, we performed conjugation experiments, and the results verified the self-transferability of pFAHZZU2591mcr-8. The genetic identity of transconjugant FAHZZU2591-*E. coli* 600 was also confirmed to be identical to *E. coli* 600 as they displayed an identical XbaI-PFGE profile (Figure S1). Interestingly, the S1-PFGE profile showed that transconjugant FAHZZU2591-*E. coli* 600 harboured two plasmids. One was similar to the donor strain, while another with a smaller size might derive from a portion of the donor plasmid (recipient *E. coli* 600 harbours no plasmid, Figure 2).

### Virulence assessment of strain FAHZZU2591

**Figure 4.**
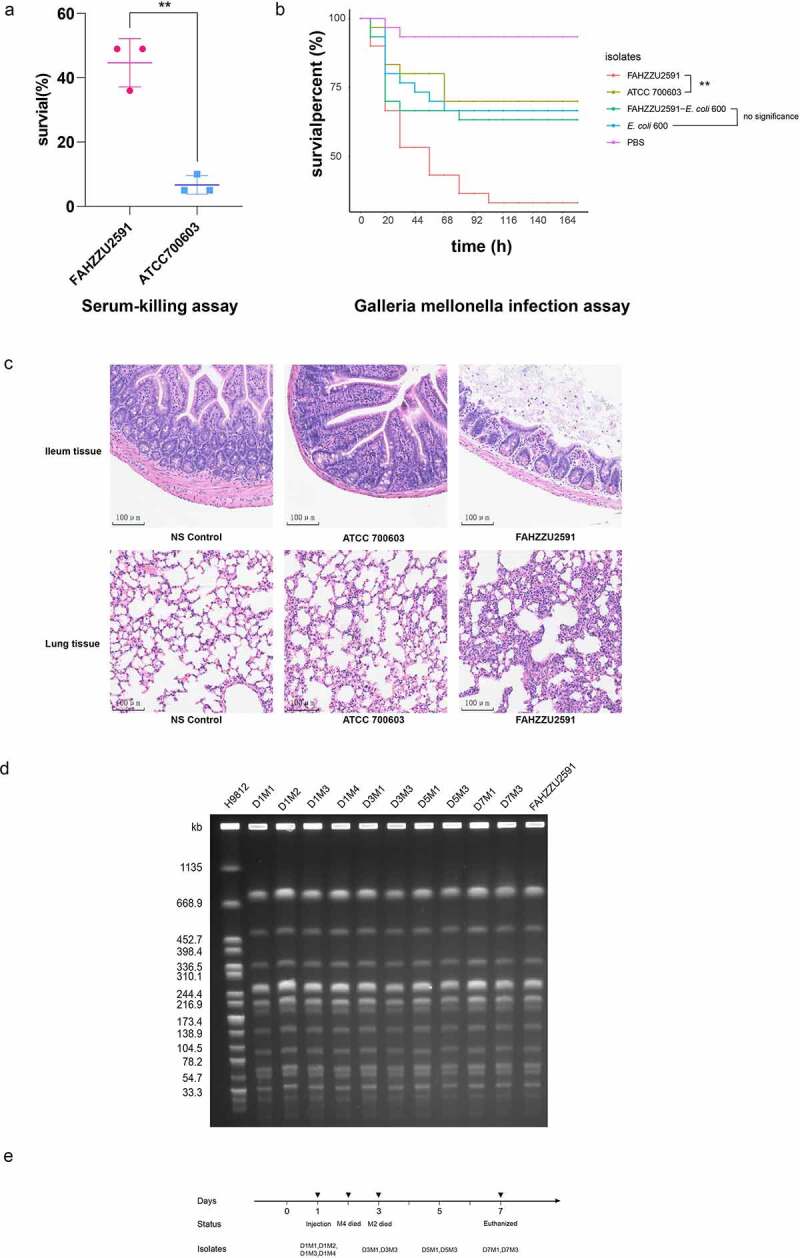
Virulence assessment of strain FAHZZU2591. (A) Serum-killing assay. Survival in a serum-killing assay of FAHZZU2591 and ATCC700603. The survival is denoted in percentage. The bars denote the means and standard errors of the mean. **P <0.01 (unpaired t test). (B) *Galleria mellonella* infection assay. Survival at 176 h in a *G. mellonella* assay of isolates FAHZZU2591, ATCC700603, transconjugant FAHZZU2591-*E. coli* 600 and recipient strain *E. coli* 600. Thirty larvae were used per strain. *G. mellonella* larvae were inoculated with 20 µl of each strain at doses of 1 × 10^6^ and incubated at 37°C; the viability was assessed over 164 h. The Kaplan-Meier estimator method was used to plot a survival curve for *G. mellonella*. The survival is denoted in percentage. Survival curves were compared with the log-rank (Mantel – cox) test. **P <0.01. (C) Haematoxylin and eosin staining of ileum and lung tissues at seven days post infection. NS: normal saline. (D) Homology analysis of *K. pneumoniae* strains isolated from faeces of mice by XbaI-PFGE. (E) Time diagram of mouse infection model assay. D represents the days of isolation, and M stands for mouse number.

Considering the emergence of *mcr-8*-bearing hvKP in the paediatric sepsis case, we assessed the virulence potential of FAHZZU2591. The serum survivability of isolate *K. pneumoniae* FAHZZU2591 was significantly stronger than the ATCC 700,603 (P < 0.01; Figure 4A). The *G. mellonella* infection assays were evaluated at 176 h after infection. Infection with FAHZZU2591 caused significantly greater mortality compared to control groups (P < 0.01). In addition, the survival rates of isolates FAHZZU2591-*E. coli* 600 and *E. coli* 600 were no obvious difference indicating plasmid pFAHZZU2591mcr-8 is not the main virulence factor of FAHZZU2591 (P > 0.05; Figure 4B). The infection model was constructed further to evaluate the pathogenicity and invasive capacity of FAHZZU2591. Two mice (M4 and M2) died on the second and third days after injection, respectively. Mice in control groups did not die until euthanized seven days later. Since day 3, the mice in the experimental group were sluggish, with dull hair and lumps on their tails while the mice in the control groups were no obvious abnormality. All of the mice were dissected, and the mice in the experimental group had plaque over their hearts and blisters in multiple organs. The ileum and lungs of mice infected with strains FAHZZU2591 and ATCC 700,603 showed tissue damage to those injected with normal saline (Figure 4C). Moreover, histopathologic examination of ileum tissue and lung tissue samples revealed greater tissue damage and infiltration of inflammatory cells in the ileum and lungs of FAHZZU2591-infected as compared to ATCC 700,603-infected mice. The basal and mucosal layers of the ileum became thinner and disorganized, the mucosal glands decreased, and the intestinal chorionic membrane shortened, and the lamina propria and inflammatory cells infiltrated. Similarly, alveolar septum thickening, alveolar oedema and hyperaemia, bronchiectasis, and leukocyte influx were observed in the lung (Figure 4C). Furthermore, based on the results of PCR, species identification and PFGE, strains isolated from the faeces of mice in the experimental group were homogenous to FAHZZU2591 which indicated it could travel through the bloodstream to the intestines (Figure 4).

### Phylogenetic analysis

**Figure 5.**
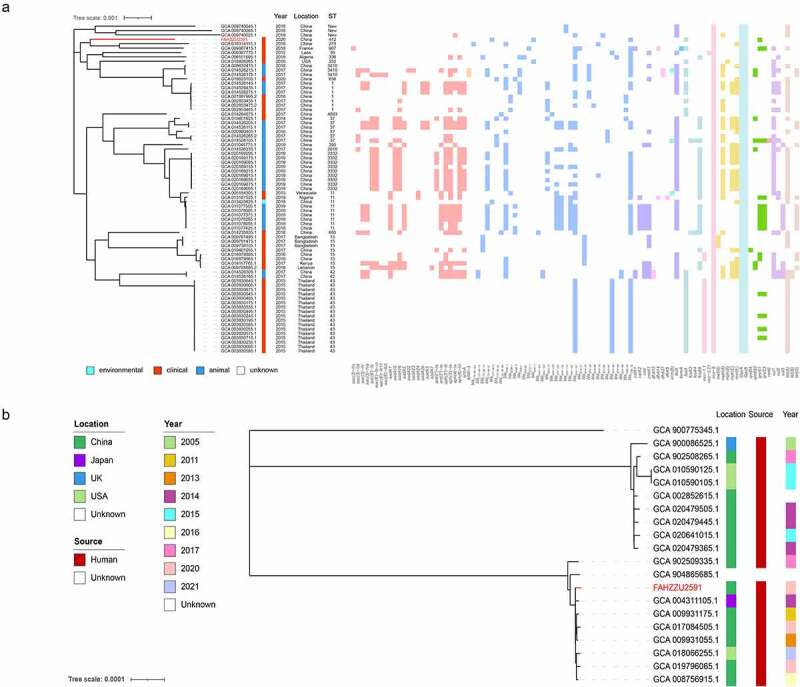
Phylogenetic analysis. (a) a phylogenetic analysis of *mcr-8*-producing *K. pneumoniae* strains based on core genomes. The drug resistance genes, collection years, locations, isolation sources, and ST types are shown. The annotation denotes the isolation sources. Different classes of resistance genes were divided into different coloured squares. (b) Phylogenetic tree of ST412 *K. pneumoniae* from NCBI database and this study. The locations, isolation sources, and collection years are shown. *K. pneumoniae* FAHZZU2591 is indicated by red.

Since the phylogenetic analysis of *K. pneumoniae* carrying the *mcr-8* gene is still lacking, we constructed a maximum likelihood phylogenetic tree that includes 75 *K. pneumoniae* with multilocus sequence types (MLST) and drug resistance genes. As shown in Figure 5 and Table S4, the most common source of *mcr-8*-bearing *K. pneumoniae* was clinical, while only one was isolated from the environment. In terms of geographical distribution, most of them were detected in China, followed by Thailand and Bangladesh, indicating Asia is the main reservoir. Resistance gene profiles showed various classes of drug-resistance genes, mainly involved in beta-lactam and aminoglycoside resistance. Further analysis revealed that all of these strains contained *oqxA* and *oqxB*. In addition, the most common MLST type among the 75 strains was ST43, while FAHZZU2591 was the only strain with the ST412 type. In addition, three new ST types were identified during the analysis. As shown in Figure 5A, FAHZZU2591 was most closely related to strain HK31 (GCA 018314115.1) and WXNZ01 (GCA 009887415.1), although they were isolated from different countries and with different ST types.

To further clarify the phylogenetic relationship of uncommon ST412 *K. pneumoniae*, a phylogenetic tree was constructed (Figure 5B). A total of nineteen *K. pneumoniae* were identified as ST412 and displayed an unexpected spatiotemporal distribution. This lineage has been persistently isolated for at least seventeen years in Asia, North America, and Europe, and all strains with definite sources were recovered from clinical.

## Discussion

*K.pneumoniae*, which acts as opportunistic pathogens, could cause several healthcare-associated infections. Among them, BSI caused by *K. pneumoniae* is the most clinical concern. BSI could lead to systemic multiple organ dysfunction syndromes (MODS), a disease that involves systemic inflammation and cell stress responses with high mortality [24,25]. Moreover, BSI is related to a more extended hospital stay and post-discharge complications, resulting in poorer outcomes [26].

Over the past two decades, the emerging hvKP as a clinically significant pathogen resulted in highly invasive infections, often complicated by devastating dissemination [13,27]. However, except for intrinsic resistance to ampicillin, hvKP rarely develops resistance to common antimicrobial agents [28]. With the sporadic spread of mobile antibiotic-resistance genes, reports involving antibiotic-resistant hvKP are increasing [29,30]. The untreatable and severe infections caused by antibiotic-resistant hvKP, significantly threaten public health. Importantly, the emergence and transmission of *mcr*-positive hvKP would become a serious public problem. Paediatric sepsis usually causes high mortality in children, and *mcr*-harbouring-hvKP-induced paediatric sepsis will make clinical treatment more difficult. Herein, we first reported an *mcr-8.2*-bearing hvKP causes paediatric sepsis. Our work revealed the unexpected presence of *mcr-8* in paediatric sepsis cases and emphasized the necessity of monitoring *mcr-8* in paediatric sepsis. The genetic environments of *mcr-8.2*-carrying plasmid were investigated, and the toxome profiles were examined. Further, we evaluated the virulence potential and performed the phylogenetic analysis of *mcr-8*-bearing *K. pneumoniae* to illustrate its global distribution.

Since the first report of hvKP in Taiwan, hvKP has been verified mainly epidemic spread in Asia [27]. Various virulence-associated features were considered significant to hvKP strains, including sequence types, capsular serotypes, and the virulence plasmid. Previous studies indicated clonal lineage CC23 was strongly associated with hvKP by conferring hypervirulence and fitness [31]. In our work, FAHZZU2591 belongs to ST412, which was a member of clonal complex 23. Additionally, the large virulence plasmid pLVPK encoding salmochelin, aerobactin, and RmpA were found to be restricted to hvKP isolates [23]. Lin *et al*. verified that the lack of pLVPK in a CC23 strain would prominently reduce virulence, proving its vital role in the hypervirulent [32,33]. Serum killing and *G. mellonella* infection assays were conducted to evaluate the virulence potential of isolate FAHZZU2591. The results presented that the virulence and fitness of FAHZZU2591 were significantly higher than the control group. In addition, *G. mellonella* infection assays in this study verified plasmid pFAHZZU2591mcr-8 is not the main virulence factor of FAHZZU2591, which is consistent with the results of toxome profile (Table 3). The high virulence of FAHZZU2591 is most likely attributed to the presence of pLVPK-like plasmid pFAHZZU2591, although direct confirmations are lacking. To further assess the pathogenicity and invasive capacity, we established mouse infection models. The results showed that multiple organ infections could be induced by the injection of FAHZZU2591 bacterial cells suspended through the tail vein. And the invasive capacity of FAHZZU2591 was more effective than ATCC 700,603. Moreover, the isolates from the faeces of mice verified strain FAHZZU2591 could enter the intestine through the bloodstream. All of those provided evidence that FAHZZU2591 was hypervirulent and highly invasive.

According to previous reports, *mcr-8* was harboured by diversified plasmids, including IncQ, IncR, IncFII, IncFIA, IncFIB, IncHI2, IncI1, IncHI1B, and IncA/C [8,34–40]. In this study, the *mcr-8*-carrying plasmid could not be classified into any known replicon type, which implies more currently unknown plasmid types may carry the *mcr-8* gene. Moreover, the transferability of pFAHZZU2591-mcr8 further promotes the horizontal transmission of *mcr-8* among different species. The NCBI database showed that pFAHZZU2591-mcr8 had the highest genetic similarity to plasmid pKP32558-2-mcr8 from a clinical *K. pneumoniae* strain with 85% coverage and 99% identity. These two plasmids harboured an identical ~94kb backbone region containing *mcr-8* and the conjugative transfer loci *tra* genes. Besides, the drug resistance region in pFAHZZU2591-mcr8 includes extended-spectrum beta-lactamases (ESBLs) genes and fluoroquinolone resistance gene (*qnrS1*) flanked by multiple transposons and ISs. Hence, we suggested that plasmid pFAHZZU2591-mcr8 and pKP32558-2-mcr8 may be derived from a common ancestor plasmid while a drug resistance region from a pJBIWA001_3-like plasmid was integrated into pFAHZZU2591-mcr8 mediated by transposons and ISs. Additionally, FAHZZU2591 carries *qnrS1* gene but is susceptible to quinolones. Unlike other *qnr* genes, an upstream sequence with a size of at least 205 bp is required for the induction of the expression of *qnrS1* [41]. Thus, we suspected that the incomplete upstream region may contribute to the lack of resistance to quinolones in FAHZZU2591.

To further understand the global distribution of *mcr-8*-bearing *K. pneumoniae*, the phylogenetic tree of *mcr-8*-positive *K. pneumoniae* was constructed. A total of 74 isolates were identified carrying *mcr-8* out of 16,472 genomes from NCBI. The sources are various and mainly focus on clinical and animals. Besides, the earliest strain was isolated in Laos in 2012 and had a close relationship with FAHZZU2591 [34]. What’s more, China is the predominant country with a high detection rate of *mcr-8*-bearing *K. pneumoniae* strains. It reminds us the persistent monitoring of *mcr-8* in China is urgent. To illustrate the co-carriage of antibiotic resistance genes in *mcr-8*-harbouring *K. pneumoniae*, the resistance gene profiles were drawn. The most common drug-resistance genes were beta-lactamases and aminoglycosides among them. The coexistence of drug-resistance genes will cause difficulties in clinical treatment which should arouse our concern and take corresponding measures.

Our study has several limitations. First, the number of *mcr*-harbouring hvKP isolates collected was not large enough and only detected one *mcr-8*-bearing hvKp. This means that the potential threat and the transmission tendency of this clone are challenging to evaluate. Second, the hospital selected in this study was limited, resulting in a potentially uneven collection of specimens. But due to the hospital in this study being the main medical institution in this province and having 12,500 beds, this limitation is compensated to some extent. Further investigations should be conducted to clarify the prevalence tendency of *mcr*-positive hvKP in clinical.

## Conclusion

Collectively, we first detected an *mcr-8.2*-harbouring hvKP isolate FAHZZU2591 causes paediatric sepsis. FAHZZU2591 was confirmed to be hypervirulent and highly invasive. Moreover, the transferability of pFAHZZU2591-mcr8 may further promote the horizontal transmission of *mcr-8* among different species. Phylogenetic analysis showed that China was the most common country with a high detection rate of *mcr-8*-bearing *K. pneumoniae* strains, and the clinic was the primary source. Our work highlights the risk of the spread of *mcr*-positive hvKP in clinical.

## Supplementary Material

Supplemental Material
