# Supplementary material for: Emergence of mcr-8.2-harboring hypervirulent ST412 Klebsiella pneumoniae strain from pediatric sepsis: A comparative genomic survey
Source: Virulence. 2022 Dec 25;14(1):233–45. doi: 10.1080/21505594.2022.2158980 (PMC9794005; doi:10.1080/21505594.2022.2158980)
Supplement: Supplemental Material [file KVIR_A_2158980_SM6793.docx]

**Table S1** PCR primers and conditions

| PCR primer | sequence |
| --- | --- |
| *mcr-1*-F | AGTCCGTTTGTTCTTGTGGC |
| *mcr-1*-R | AGATCCTTGGTCTCGGCTTG |
| *mcr-2*-F | CAAGTGTGTTGGTCGCAGTT |
| *mcr-2*-R | TCTAGCCCGACAAGCATACC |
| *mcr-3*-F | AAATAAAAATTGTTCCGCTTATG |
| *mcr-3*-R | AATGGAGATCCCCGTTTTT |
| *mcr-4*-F | TCACTTTCATCACTGCGTTG |
| *mcr-4*-R | TTGGTCCATGACTACCAATG |
| *mcr-5*-F | ATGCGGTTGTCTGCATTTATC |
| *mcr-5*-R | TCATTGTGGTTGTCCTTTTCTG |
| *mcr-6*-F | AGCTATGTCAATCCCGTGAT |
| *mcr-6*-R | ATTGGCTAGGTTGTCAATC |
| *mcr-7*-F | GCCCTTCTTTTCGTTGTT |
| *mcr-7*-R | GGTTGGTCTCTTTCTCGT |
| *mcr-8*-F | TCAACAATTCTACAAAGCGTG |
| *mcr-8*-R | AATGCTGCGCGAATGAAG |
| *mcr-9*-F | TTCCCTTTGTTCTGGTTG |
| *mcr-9*-R | GCAGGTAATAAGTCGGTC |
| *mcr-10*-F | GGACCGACCTATTACCAGCG |
| *mcr-10*-R | GGCATTATGCTGCAGACACG |

PCR reaction system

| ingredient | Volume (μL) |
| --- | --- |
| 2 × Taq Master Mix enzyme | 22 |
| primer-F | 1 |
| primer-R | 1 |
| DNA | 1 |
| ddH_2_O | 25 |
| Total | 50 |

PCR reaction conditions (*mcr1* to *mcr5*)

| Temperature | Time | Cycle |
| --- | --- | --- |
| 94℃ | 15min | 1 |
| 94℃ | 30s | 25 |
| 58℃ | 90s | 25 |
| 72℃ | 60s | 25 |
| 72℃ | 10min | 1 |
| 4℃ | 1h | 0 |

PCR reaction conditions (*mcr6* to *mcr10*)

| Temperature | Time | Cycle |
| --- | --- | --- |
| 95℃ | 5 min | 1 |
| 95℃ | 1 min | 30 |
| 54℃ | 30 s | 30 |
| 72℃ | 30 s | 30 |
| 72℃ | 7 min | 1 |
| 4℃ | 1 h | 0 |

**Table S2** The primer sequences for the probe of Southern blotting

| Primer | Sequence |
| --- | --- |
| *mcr-8*-F | TCAACAATTCTACAAAGCGTG |
| *mcr-8*-R | AATGCTGCGCGAATGAAG |
| *rmpA*-F | TTTACCGTGATTGATTGAATTTTT |
| *rmpA*-R | TTGCGTCTATTCATCGCTTTT |
| *rmpA2*-F | CCATGCAAACACAAACACAA |
| *rmpA2*-R | TTCTCAACCTCCTTCTTTGAGC |

**Table S3** Genomic features of *K. pneumoniae* FAHZZU2591

| Feature | Chromosome | pFAHZZU2591 | pFAHZZU2591mcr-8 |
| --- | --- | --- | --- |
| Size (bp) | 5,236,068 | 202,973 | 110,627 |
| G + C content (%) | 57.6 | 49.9 | 52.0 |
| No. of protein-coding sequences | 4,787 | 199 | 129 |
| No. of tRNA genes | 85 | 0 | 0 |
| No. of rRNA genes | 25 | 0 | 0 |
| Accession numbers | CP083751 | CP083752 | CP083753 |

**Table S4** Characteristics of 75 *mcr-8*-harboring *K. pneumoniae* genome sequences analyzed in this study. The data were downloaded from NCBI database

| Strain | Location | Year | Source | ST |
| --- | --- | --- | --- | --- |
| GCA_009740045.1 | China | 2018 | unknown | new |
| GCA_009740085.1 | China | 2018 | unknown | new |
| GCA_009740025.1 | China | 2018 | unknown | new |
| FAHZZU2591 | China | 2020 | clinical | 412 |
| GCA_018314115.1 | China | 2016 | clinical | 273 |
| GCA_009887415.1 | France | 2018 | clinical | 967 |
| GCA_009387775.1 | Laos | 2012 | clinical | 39 |
| GCA_006151695.1 | Algeria | 2018 | clinical | 336 |
| GCA_019928265.1 | USA | 2016 | clinical | 252 |
| GCA_008632415.1 | China | 2018 | animal | 3410 |
| GCA_014526215.1 | China | 2017 | animal | 3410 |
| GCA_014526175.1 | China | 2017 | animal | 3410 |
| GCA_018623105.1 | China | 2020 | clinical | 656 |
| GCA_014526145.1 | China | 2017 | animal | 1 |
| GCA_014529435.1 | China | 2017 | animal | 1 |
| GCA_014526275.1 | China | 2017 | animal | 1 |
| GCA_001887995.2 | China | 2016 | clinical | 1 |
| GCA_002853455.1 | China | 2017 | clinical | 1 |
| GCA_002853475.2 | China | 2017 | clinical | 1 |
| GCA_002853465.1 | China | 2017 | clinical | 1 |
| GCA_014284075.1 | China | 2017 | clinical | 4683 |
| GCA_019401625.1 | China | 2014 | clinical | 37 |
| GCA_014526205.1 | China | 2017 | animal | 37 |
| GCA_014526115.1 | China | 2017 | animal | 37 |
| GCA_020990405.1 | China | 2016 | animal | 37 |
| GCA_014526265.2 | China | 2017 | animal | 37 |
| GCA_014526105.1 | China | 2017 | animal | 37 |
| GCA_011045775.1 | China | 2019 | animal | 395 |
| GCA_014526235.1 | China | 2017 | animal | 2018 |
| GCA_020169295.1 | China | 2019 | animal | 3332 |
| GCA_020169175.1 | China | 2019 | animal | 3332 |
| GCA_020169095.1 | China | 2019 | animal | 3332 |
| GCA_020169155.1 | China | 2019 | animal | 3332 |
| GCA_020169215.1 | China | 2019 | animal | 3332 |
| GCA_020169015.1 | China | 2019 | animal | 3332 |
| GCA_020169035.1 | China | 2019 | animal | 3332 |
| GCA_020169075.1 | China | 2019 | animal | 3332 |
| GCA_020169055.1 | China | 2019 | animal | 3332 |
| GCA_005184305.1 | Venezuela | 2015 | clinical | 11 |
| GCA_013187525.1 | Nigeria | 2019 | clinical | 11 |
| GCA_013423825.1 | China | 2018 | environmental | 11 |
| GCA_011077505.1 | China | 2019 | animal | 11 |
| GCA_011078065.1 | China | 2018 | animal | 11 |
| GCA_011077375.1 | China | 2018 | animal | 11 |
| GCA_011078265.1 | China | 2019 | animal | 11 |
| GCA_011078055.1 | China | 2018 | animal | 11 |
| GCA_011077425.1 | China | 2018 | animal | 11 |
| GCA_014235835.1 | China | 2016 | clinical | 685 |
| GCA_009791495.1 | Bangladesh | 2017 | clinical | 15 |
| GCA_009791475.1 | Bangladesh | 2017 | clinical | 15 |
| GCA_009734105.1 | Bangladesh | 2017 | clinical | 15 |
| GCA_019401055.1 | China | 2017 | clinical | 15 |
| GCA_016879895.1 | China | 2016 | clinical | 15 |
| GCA_016879865.1 | China | 2016 | clinical | 15 |
| GCA_014117765.1 | Kenya | 2017 | clinical | 15 |
| GCA_009795695.2 | Lebanon | 2018 | clinical | 15 |
| GCA_014526305.1 | China | 2017 | animal | 42 |
| GCA_014526165.1 | China | 2017 | animal | 42 |
| GCA_003830645.1 | Thailand | 2015 | clinical | 43 |
| GCA_003830605.1 | Thailand | 2015 | clinical | 43 |
| GCA_003830675.1 | Thailand | 2015 | clinical | 43 |
| GCA_003830545.1 | Thailand | 2015 | clinical | 43 |
| GCA_003830485.1 | Thailand | 2015 | clinical | 43 |
| GCA_003830175.1 | Thailand | 2015 | clinical | 43 |
| GCA_003830535.1 | Thailand | 2015 | clinical | 43 |
| GCA_003830495.1 | Thailand | 2015 | clinical | 43 |
| GCA_003830245.1 | Thailand | 2015 | clinical | 43 |
| GCA_003830195.1 | Thailand | 2015 | clinical | 43 |
| GCA_003830585.1 | Thailand | 2015 | clinical | 43 |
| GCA_003830255.1 | Thailand | 2015 | clinical | 43 |
| GCA_003830575.1 | Thailand | 2015 | clinical | 43 |
| GCA_003830715.1 | Thailand | 2015 | clinical | 43 |
| GCA_003830235.1 | Thailand | 2015 | clinical | 43 |
| GCA_003830695.1 | Thailand | 2015 | clinical | 43 |
| GCA_003830595.1 | Thailand | 2015 | clinical | 43 |

**
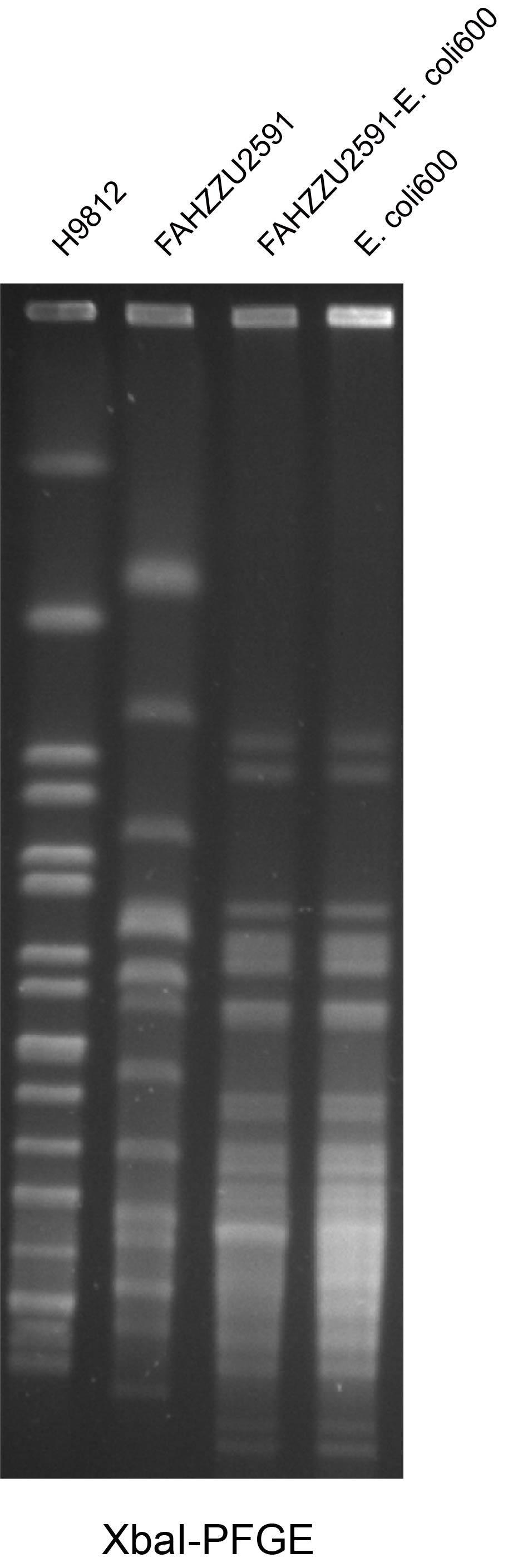
**

**Figure S1** The genetic identity of transconjugant FAHZZU2591-*E. coli* 600 by XbaI-PFGE.
